# Not All Spikes Are Equal

**DOI:** 10.3390/jcm14228071

**Published:** 2025-11-14

**Authors:** Anita N. Datta

**Affiliations:** Department of Pediatrics, Division of Neurology, BC Children’s Hospital, Faculty of Medicine, University of British Columbia Vancouver, 4480 Oak Street, Vancouver, BC V6H 3N1, Canada; anita.datta@cw.bc.ca; Tel.: +1-604-875-2345; Fax: +1-604-875-2285

**Keywords:** children, EEG, refractory epilepsy, neurodevelopmental disorders, non-invasive, EEG

## Abstract

EEG remains the primary diagnostic tool for evaluating seizures in children, with interictal epileptiform discharges (IEDs) serving as key biomarkers of epileptogenic activity. However, not all IEDs have the same prognostic significance. Variations in IED topography, morphology, frequency, and timing influence outcomes in pediatric epilepsy. The developing brain’s maturation affects IED location and features, creating age-specific patterns with distinct implications. For example, occipital and midline IEDs are common in young children, with midline IEDs strongly linked to increased seizures and developmental delay than control patients. Morphological features provide additional prognostic stratification. While centrotemporal IEDs with tangential dipoles are well-established as favorable prognostic markers, IEDs exhibiting tangential dipoles in any brain region are associated with more benign clinical courses than control patients. Conversely, positive sharp waves persisting beyond the neonatal period signal less favorable prognosis, including developmental delay, abnormal neurological examination, and structural brain abnormalities. Additionally, IEDs occurring on ripples have been shown to serve as more reliable interictal biomarkers of the epileptogenic zone than IEDs or ripples alone. Topography, frequency and sleep-state dependence also carry clinical significance, as frequent IEDs during slow-wave sleep may impact cognition. Furthermore, the temporal context of IED occurrence during seizure onset, treatment, activation procedures, medication withdrawal, or after epilepsy surgery provides valuable prognostic information. Recognition of these nuanced electrophysiological distinctions enhances clinicians’ ability to predict clinical trajectories and optimize long-term management strategies.

## 1. Introduction

Since its introduction in 1929 by German psychiatrist Hans Berger [1], routine EEG is a fundamental diagnostic tool for evaluating epileptic seizures, with interictal epileptiform discharges (IEDs) serving as a key diagnostic marker. IEDs are distinctive complexes from background activity and are observed when seizures are not actively occurring. The neurophysiological basis of focal IEDs involves the synchronized depolarization of cortical pyramidal neurons, generating vertically oriented dipoles perpendicular to the cortical surface. Scalp EEG IEDs and ictal discharges reflect cortical generators that encompass an area of approximately 10 to 20 cm^2^, corresponding to a sub-lobar region [2]. IEDs differ from background rhythms, reflecting abnormal hyper-synchronous excitability [3,4]. Identification of IEDs can aid in predicting seizure recurrence risk, localizing potential epileptogenic foci, and identifying epilepsy syndromes. However, interictal epileptiform discharges (IEDs) are not definitive markers of epilepsy, and their usefulness for localization has significant limitations.

This review will examine the clinically significant features of focal IEDs observed on routine EEG in children, emphasizing how specific characteristics may indicate pathological significance. Key features to be discussed include topographic distribution, morphology, discharge frequency, patient age, and timing relative to clinical presentation. These IED characteristics will be analyzed in relation to important clinical outcomes, including the presence or absence of epilepsy, drug-resistant epilepsy, developmental delay, seizure etiology, neuroimaging abnormalities, and concordance with the seizure-onset zone in epilepsy surgery patients. By examining these relationships, this review aims to delineate which features of IED are associated with more severe clinical presentations and which are linked to more favorable outcomes.

## 2. Significance of IED Location and Topography

Focal IEDs can are observed in the frontal, temporal, central, parietal, occipital, or midline areas, and their topography can occasionally provide information related to seizure focus and prognosis. These findings have important implications for interpreting scalp-recorded IEDs.

However, it is important to note that scalp EEG can only detect IEDs reaching the cortical surface with sufficient spatial extent and synchronization, potentially missing deeper generators or small foci. Furthermore, IEDs visualized on scalp EEG may represent propagated activity from distant generators rather than the true epileptogenic onset zone [3]. Field spread, volume conduction effects, and limited spatial resolution inherent to scalp recordings mean that spike localization should be presented as an approximation rather than precise delineation of epileptogenic tissue recordings [3]. Bone, dura and scalp tissue attenuate EEG signals, further hampering the sensitivity of scalp recordings [5]. For example, mesial temporal IEDs do not necessarily indicate temporal lobe epilepsy, as EEGs in patients with extratemporal epilepsies also have a tendency to exhibit temporal IEDs [6]. Also, frontal lobe discharges can be particularly difficult to distinguish due to field spread and overlapping voltage fields.

The lack of IED spatial sensitivity on scalp EEG was demonstrated in a study evaluating the localization of ictal and IEDs in patients with clearly defined lesions on MRI, which varied by brain region. EEGs in patients with central (46.7%) and frontal (17.8%) lesions more often lacked IEDs compared to patients with temporal (4.2%) or parieto-occipital (7.0%) lesions. IEDs were found exclusively in the lesioned lobe in 45.6% of temporal, 31.3% of central, 25% of frontal, and only 5% of parieto-occipital lesions. Furthermore, ictal EEG changes in the lesioned lobe occurred more commonly in temporal (58%), frontal (43.8%), and central (42.1%) than in parieto-occipital (28.9%) lesions (*p* < 0.05) [7]. Despite these challenges with the limitations of localization of IEDs on scalp EEG, various studies have looked at the significance of IED location on scalp EEG in relation to clinical features in children.

### 2.1. Centrotemporal

Centrotemporal IEDs in children can represent a benign functional focus, including those observed in self-limited epilepsy with centrotemporal spikes (SeLECTS) [8]. Also, not all centrotemporal spikes are associated with clinical seizures and they are present in approximately 15% of seizure-free siblings with SeLECTS, suggesting a genetic susceptibility [9]. Centrotemporal IEDs can also occur in less favorable contexts, such as in children with brain injury [10], and those with temporal or focal motor epilepsy [11]. When centrotemporal IEDS are encountered, the ability to predict prognosis is of paramount importance.

**Figure 1:**
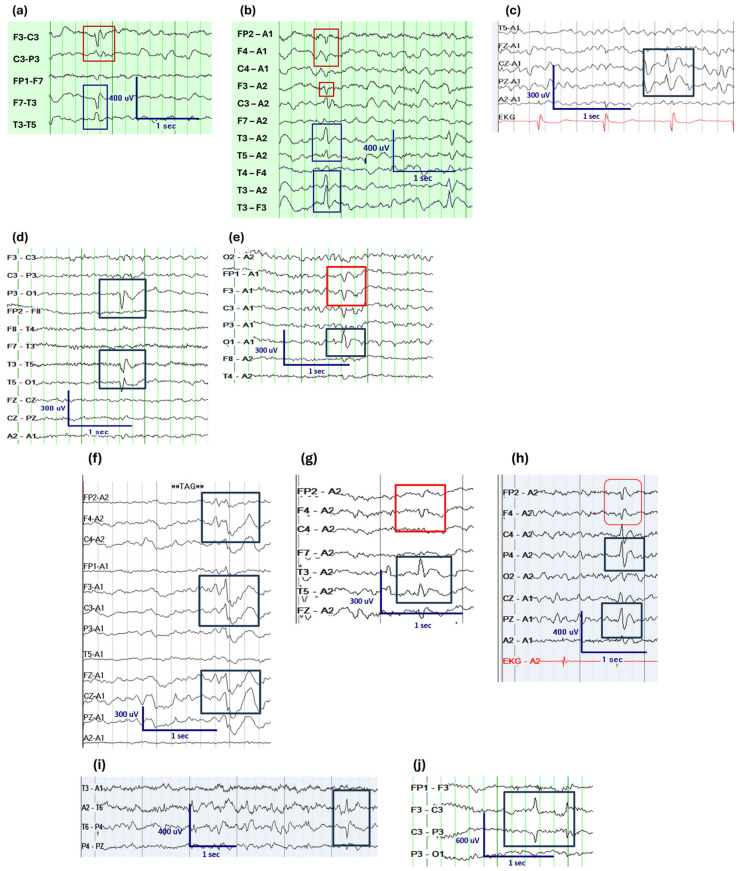
Examples of IEDs with Varying Prognostic Significance. (**a**) Central Temporal IEDs (C4/T4) in bipolar montage. Paper speed 30 mm/s. (**b**) Central Temporal IEDS (C4/T4) in referential montage showing tangential dipole with simultaneous and temporal negativity (blue box) and frontal positivity (red box). Paper speed 30 mm/s. (**c**) Midline IED with midline bipolar montage. Paper speed 30 mm/s. (**d**) IED with occipital negativity (O1) in bipolar Montage. Paper speed 30 mm/s. (**e**) IED with occipital negativity (O1) (blue box) and simultaneous frontal positivity (F4) (red box) in referential montage. Paper speed 30 mm/s. (**f**) Hypnagogic sharply contoured waves (HFSC) in referential montage. Paper speed 30 mm/s. (**g**) IED with tangential dipole with simultaneous temporal negativity (T3/T5) (blue box) and frontal positivity (Fp2/F4) (red box). Paper speed 30 mm/s. (**h**) IED with tangential dipole with simultaneous parietal negativity P4/PZ (blue box) and frontal positivity (Fp2/F4) (red box). Paper speed 30 mm/s. (**i**) Positive IED (T6) in bipolar montage. Paper speed 15 mm/s. (**j**) Positive IED (C3) in bipolar montage. Paper speed 30 mm/s.

In SeLECTS, centrotemporal IEDs are typically high-amplitude, unilateral or independent bilateral diphasic IEDs located in the central or mid-temporal region, potentiated by drowsiness and sleep [12]. They are age-specific, rarely occurring before age 3 or after age 16 years [13]. Notably, centrotemporal IEDs with tangential dipoles, exhibiting simultaneous frontal positivity and central/temporal negativity, are highly predictive of a more favorable prognosis (Figure 1a,b) [14]. Conversely, IEDs associated with cortical lesions tend to be lower in voltage, sharper, have more uniform morphology [15], lack age specificity, and do not display tangential dipoles [14]. The mechanisms of generation of tangential dipoles are discussed in the section on IED morphology.

### 2.2. Midline

Midline IEDs are characterized by cortical generators originating from the midsagittal brain regions, specifically at electrode sites Fz, Cz, and Pz, corresponding to the midfrontal, midcentral, and mid-parietal regions (Figure 1c). Midline IEDs are more common in young children, with a mean age range of 2.5 to 6 years [16,17,18,19,20,21,22,23,24,25]. Midline IEDs are strongly associated with seizures [16,17,18,19,20,21,22,23,24,26,27]. The etiology of midline IEDs is heterogeneous; however, they have been linked to abnormalities in subcortical gray or white matter [27]. Furthermore, in comparison to control patients undergoing EEGs at a tertiary care pediatric center, midline IEDs are associated with an increased risk of developmental delay, which is likely indicative of underlying brain dysfunction and etiology, rather than a direct causative factor [27].

### 2.3. Occipital

Occipital IEDs are age-dependent, seen in 0.9% of normal preschool age and 0.1% of school-aged children [28,29,30], underscoring their developmental significance. These IEDs can also be detected in individuals without a history of seizures. Specifically, sharp, narrow IEDs resembling EMG activity or needle morphology IED, can occur in children with congenital blindness [31]. In most children with occipital IEDs, underlying structural abnormalities, such as periventricular leukomalacia or metabolic abnormalities, such as mitochondrial encephalomyopathy, lactic acidosis and stroke-like episodes, are identified [32]. However, a smaller subset presents with self-limited epilepsy syndromes, including SeLEAS (Self-Limited Epilepsy with Autonomic Symptoms) and COVE (Childhood Occipital Visual Epilepsy). Predictors of these self-limited epileptic syndromes include the presence of generalized spike-and-wave discharges or a normal background activity [32]. Additionally, a study identified that a tangential dipole pattern, characterized by occipital negativity and frontal-central positivity, along with a history of abnormal ictal eye movements, predicted of self-limited epilepsy (Figure 1d,e) [30].

### 2.4. Frontal

Frontal IEDs typically demonstrate maximal negativity over frontopolar, frontotemporal, or anterior midline regions. However, compared to epilepsy originating from other brain regions, such as the temporal lobe, IEDs are detected less frequently in frontal lobe epilepsy. This reduced detection rate occurs predominantly with basal frontal foci, including the orbitofrontal cortex, and mesial or interhemispheric regions, such as the supplementary motor cortex. These areas generate IEDs that are poorly recorded on scalp EEG because the orientation of the spike dipole must be approximately orthogonal to the scalp surface for detection and dipoles oriented parallel to the scalp remain undetectable by scalp electrodes [3]. Furthermore, scalp detection of frontal IEDs is often limited by rapid propagation and artifact contamination, particularly from eye movement and muscle activity. A study demonstrated that children with a first unprovoked seizure with frontal IEDs on initial EEG may be at higher risk for the development of epilepsy later in life than those with IEDs in other regions [33].

Hypnagogic sharply contoured waves (HFSC) are defined as isolated sharply contoured waves, with or without a slow component, lasting 0.25 to 0.5 s, maximal in independent, bilateral or midfrontal regions (Fz, F3-4, and Fp1-2), which borders on epileptiform but does not meet the criteria for IED (Figure 1f). HFSC occurs in drowsiness, persists into stage two sleep, coexists with normal sleep architecture, and may become sharper in morphology with sleep progress [34]. There is often debate whether these waveforms represent hypnagogic hypersynchrony or atypical sleep potentials (“frontal K-complexes”). However, the frontal predominance is always of concern. In a study, when HFSC was compared to children with normal EEGs, bifrontal slowing, and generalized spike wave discharges, it was observed that the presence of HFSC is associated with a higher risk of seizures, epilepsy, and developmental delay relative to children with normal EEGs. Also, the HFSC group had more developmental delay than the normal EEG group, but similar to the GSW group [34]. Therefore, this can be another potential prognostic finding when encountered on routine pediatric EEG.

### 2.5. Measurable Effects of Focal IEDs

It has been demonstrated in various studies that focal IEDs impact cognition. For example, a combined MEG/EEG study in children with self-limited epilepsies observed that children with left centrotemporal IEDs performed worse with complex language tasks, and children with occipital IEDs performed significantly lower in information processing, especially in visual transformation tasks on neuropsychological testing [35]. However, other studies utilizing scalp EEG have found little or no relationship between IED laterality and cognition [36,37,38,39,40,41]. Another example is a study of children with SeLECTS, where IEDs and not seizures were shown to trigger reorganization of the laterality of speech perception, resulting in a bilateral representation of auditory and verbal stimuli and a loss of the usual left hemisphere advantage in processing these stimuli [42].

These discrepancies may reflect several important factors. First, MEG demonstrates superior sensitivity in detecting cortical activity compared to scalp EEG, particularly from tangentially oriented sources [43]. Second, differences in patient age and developmental stage at the time of assessment may influence results. For example, IED frequency, lateralization, and associated cognitive and behavioral symptoms may evolve over the course of epilepsy, and this temporal variability was not measured in some studies. Third, variability in the timing and frequency of IEDs during cognitive testing can affect detection of their impact. Fourth, methodological differences in neuropsychological assessment tools across studies may contribute to inconsistent findings. For example, some testing protocols may not have detected more subtle abnormalities, such as oromotor or handwriting dysfunction, which have been previously described in these populations [44,45].

Focal IEDs may also result in transient cognitive impairment, occurring at the time of the IED. By means of a computerized system of IED, presentation of visual stimuli, and registration of reaction times, Shewmon et al. demonstrated that focal posterior IEDs cause transiently prolonged reaction times and increased nonperception and misperception of stimuli, especially contralateral to the IED [46,47,48]. Also, it was noted that the following slow wave, with surround hyperpolarization, transiently disrupts aspects of cortical functioning, in addition to whatever effect the IED itself may have. Furthermore, in patients undergoing scalp video-EEG monitoring, Binne et al. observed transient deficits in spatial tasks linked to the occurrence of right-sided IEDs and verbal tasks to left-sided IEDs [49]. Despite these findings, treatment with anti-seizure medications is focused on controlling seizures rather than targeting focal IEDs, apart from cases of epileptic encephalopathy.

## 3. Significance of IED Morphology

On the EEG, IEDs are transient potentials, distinguishable from the background, with a sharp negative peak. A spike has a duration of 20 to 70 ms with a more rapid ascending than descending peak, whereas a sharp wave has a duration of 70 to 200 ms [50]. The International Federation of Clinical Neurophysiology (IFCN) has proposed six operational criteria for defining IEDs based on morphological features including sharp or spiky morphology, different wave durations than the background activity, waveform asymmetry, after-going slow wave, disruption of background activity, and a distribution suggestive of a cerebral source [51]. These criteria in sensor space and analysis in source space have high specificity (>95%) and sensitivity (81–85%) for identification of true IEDs, helping to distinguish pathological IEDs from normal sharp transients that are often misread [51].

Studies have shown that the morphology of IEDs varies with age. Focal IEDs tend to become more prevalent as children grow older, and their electrophysiological characteristics evolve. With maturation, IEDs tend to appear less sharp, with lower amplitudes, and less prominent slow-wave components. Furthermore, with age, the localization of IEDs becomes more lateralized, with a left-sided preponderance [52]. Previous studies have also examined spike asymmetry, where the duration of the first half-wave is divided by the duration of the second half-wave, as defined by Henze et al. [53]. A value less than 1 corresponds to a shorter duration of the first half-wave compared to the second half-wave. Notably, spike asymmetry remains consistent across different ages, regardless of developmental stage [52].

### 3.1. Tangential Dipole

On EEG, tangential generators often originate from sulcal walls, comprising 70% of the cortical surface [54], and the mesial cortex, such as basal temporal cortices. However, tangential generators in adjacent fissural walls can distort scalp potential fields, leading to the predominance of radial potentials from gyri crowns [55]. As discussed previously, it is well established that IEDs with tangential dipole configurations in the centrotemporal regions are associated with SeLECTS, which has known favorable outcomes [14]. Tangential dipoles are observed in SeLECTS because the seizure generator is located in the lower part of the peri-rolandic region, near the upper Sylvian fissure close to the central sulcus, which governs orofacial movements and sensation. The horizontal orientation of this dipole results from the tangential alignment of the cortical source relative to the scalp surface. The neuronal generator is positioned such that current flows horizontally from the centrotemporal region (negative pole) toward the frontal region (positive pole), rather than vertically, which would produce a radial dipole [14].

Tangential dipoles in other regions are less common (Figure 1d,g,h). However, a study evaluated children with focal IEDs with tangential dipoles in the frontal, temporal, central, parietal and occipital regions and observed that the 232 of 898 children with tangential dipoles have significantly better clinical outcomes in comparison to patients without tangential dipole IEDs. Specifically, IEDs with tangential dipoles were linked to lower rates of drug-resistant epilepsy, developmental delay, school-performance issues, autism, and abnormal exams, regardless of brain region [56]. These findings were also observed on logistic regression analysis within the different brain regions. However, limitations of this study include its focus on group-level analysis, without accounting for other clinical factors. For example, due to insufficient power, it could not be determined if tangential dipole IEDs were associated with better outcomes in patients with similar seizure etiologies. However, despite the study limitations, clinicians can be aware that overall, the presence of IEDs with tangential dipoles on routine EEG, regardless of location, is suggestive of a more favorable prognosis. A potential explanation for the observation that sulcal generators are associated with a more benign epileptic course is rooted in the anatomical and functional differences between gyri and sulci. These differences include variations in neuronal populations and their interconnections [57,58]. Specifically, gyri comprise a thicker cortex [59], better formed laminae, denser vascularization and pyramidal arborisation in comparison to sulci [60,61]. Additionally, lower connectivity patterns in sulci have been observed compared to gyri, with variations in long- and short-distance connecting fibers that facilitate intra-lobar and cross-hemispheric communication [57,62,63]. These structural and functional distinctions may contribute to the differing clinical courses observed in epilepsy related to sulcal versus gyral generators. Tangential dipoles are primarily observed in children, compared to adults, due to ongoing neurodevelopmental processes including synaptogenesis, synaptic pruning, myelination, and gray matter reduction. These changes alter brain surface morphology and gyral-sulcal architecture, which may influence regional neuronal connectivity patterns [64,65].

### 3.2. Positive IEDs

Positive spike wave discharges (PSW) on EEG are common in neonates. This increased prevalence is primarily attributed to incomplete neuronal migration, immature myelination, and the underdevelopment of dendrites in pyramidal cells, apart from apical dendrites [66]. Although common in neonates, PSW occur less commonly in children (Figure 1i,j). PSW have been linked to underlying chronic or static lesions, particularly malformations of cortical development, a remote history of hydrocephalus or periventricular leukomalacia, which may also result in deep excitatory generators [67,68,69]. Disruptions in neuronal migration may lead to the presence of heterotopic neurons in the subcortical regions. This intricate network of neurons may affect the extracellular potential fields associated with EPSPs, which are generated by afferent fibers positioned in the superficial layers of the apical dendrites of excitatory neurons. PSW may also arise in patients with post-operative or traumatic injuries, where anatomical cortical changes and bone defects, may result in detection of positivity at the skull surface. A large retrospective study observed that clinically, PSWs are associated with higher risks of seizures, epilepsy, drug-resistant epilepsy, developmental delays, academic difficulties, and abnormal neurological examinations when compared to children with negative IEDs. These outcomes likely reflect underlying structural cortical abnormalities rather than the positivity of the IEDs themselves [67,68]. However, study limitations include that it was a retrospective study and looked at children on a group level, rather than stratifying by other clinical risk factors.

## 4. IED Frequency

**Figure 2:**
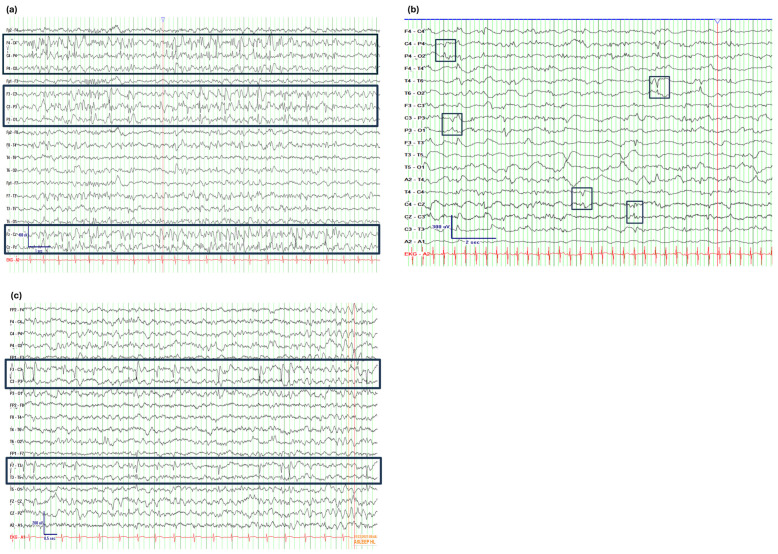
Examples of EEGs with Frequent IEDs and Their Clinical Significance Across Different Contexts. (**a**) 7-year old female with new-onset nearly continuous right and left central parietal IEDs during slow wave sleep in bipolar montage (depicted in the boxes). Paper speed 15mm/s. EEG findings corresponded to new-onset expressive language regression. (**b**) Term 10-day old neonate with excessive multifocal central/temporal/parietal sharp transients in bipolar montage (examples depicted in the boxes). Paper speed 15 mm/s. The EEG was conducted while the neonate was experiencing sepsis. The patient had no history of seizures and did not develop any seizures during a five-year follow-up period. (**c**) 5-year old female with Rett syndrome and MECP2 mutation. EEG demonstrates frequent left central (C3) and temporal (T3) IEDs in sleep in bipolar montage (depicted in the boxes). Paper speed 15 mm/s. The child does not have a history of seizures.

Some children can have marked IED activation in sleep accompanied by cognitive, language, behavioral, and motor regression, consistent with Developmental and Epileptic Encephalopathy with Spike Wave Activation in Sleep (DEE-SWAS) [8]. IED activity tends to be diffuse but can also be focal or multifocal, particularly in the frontal regions [70]. Previously, DEE-SWAS was defined as IEDs that comprise >85% of slow wave sleep; however, it is now established that lower percentages of IEDs in sleep may be associated with cognitive regression or behavioral function. The IED topography, duration of the DEE-SWAS pattern, and underlying etiology influence the severity and nature of cognitive dysfunction in affected children. Landau–Kleffner syndrome (LKS) is a specific subtype of DEE-SWAS, where regression affects mainly language, with an acquired auditory agnosia. The LKS EEG usually has IED foci in the posterior temporal or parietooccipital regions, which are potentiated in sleep [71]. In addition to SeLECTs and SeLEAS, other focal epilepsies may evolve to DEE-SWAS associated with neuropsychological regression, especially in verbal and visual tasks that impact academic and familial functioning (Figure 2a). High IED burden on EEG is shown to correlate with poorer outcomes in visual perception, fine motor performance and short-term memory, implicating that IEDs may alter the cerebral mechanisms underlying cognitive functioning [72]. Importantly, early diagnosis and timely intervention of DEE-SWAS are crucial, as they are significantly linked to better prognosis and can help prevent long-term developmental sequelae [73]. However, it is important to be aware that some children with genetic or structural etiologies may have intellectual impairment and frequent IEDs without a history of clear regression. In these cases, the IEDs may be just a manifestation of the underlying pathologic process rather than the cause of cognitive impairment.

## 5. Significance of Timing of IEDs

The timing of IEDs relative to age is of clinical significance, as the implication of IEDs in neonates and infants is different from that of school-aged children and adolescents.

IEDs differ from background rhythms, reflecting abnormal hyper-synchronous excitability [3,74]. With brain maturation, pediatric EEGs and IEDs evolve over time. Understanding normal EEG developmental patterns is essential for understanding their clinical significance. In full-term newborns, frontal, central and temporal occasional sharp transients are common non-pathological findings during wakefulness, active sleep, and within tracé alternant bursts during quiet sleep, typically disappearing by 4 to 6 weeks of age [75].

Excessive bilateral sharp transients can be an indicator of an encephalopathy of non-specific etiology, while persistent localized sharp transients may represent an epileptic focus [5].

With brain development, the location and characteristics of pathological IEDs also demonstrate age-dependent patterns. Midline IEDs, occipital and parietal IEDs, are most frequently observed in younger children 2.5 and 6 years [24,25,27]. Over time, IEDs tend to decline or shift anteriorly toward the mid-temporal regions [76]. Focal IEDs become more prevalent with age, more lateralized and their electrophysiological characteristics evolve, appearing less sharp, with lower amplitudes and less prominent slow-wave components [52]. Age-related patterns also influence epilepsy syndrome presentation, as certain syndromes emerge and remit at specific developmental stages. For example, self-limited epilepsy with autonomic seizures (SeLEAS) typically occurs in children aged 3–6 years, while childhood occipital visual epilepsy (COVE) manifests in older school-aged children [8]. Compared to neonates, infants, and elementary-age children, older children and adolescents with persistent IEDs are more likely to have ongoing epileptogenicity and face a higher risk of seizures [76].

### 5.1. Neonatal IEDs

In neonates and infants, IEDs often reflect the immature state of cortical development and are not necessarily a predictor for future development of epilepsy. Studies have confirmed that neonatal IEDs, or sharp transient discharges, are not reliable predictors of adverse clinical outcomes, including seizures, developmental delay or abnormal neurological examination (Figure 2b) [77,78,79]. In some studies, positive rolandic sharp waves were correlated with the occurrence of intraventricular hemorrhage and white matter injury [80,81,82]. However, these retrospective studies have several limitations, including short follow-up durations and small cohort sizes, with most involving fewer than 100 patients, a mixed population of premature and term neonates, lack of standardized interpretation of EEG findings, limited information on neurological outcomes, and a small number of cases with specific abnormal findings. Conversely, larger retrospective studies focused on a homogeneous group of term neonates with extended follow-up periods and assessed various clinical outcome parameters have not found that the presence of positive rolandic IEDs predicts clinical prognosis in term neonates [79,83]. In neonates, ictal discharges are correlated with unfavorable neurological outcomes and the development of epilepsy. Additionally, abnormal EEG background and background suppression are associated with abnormal neurological examinations, developmental delay and epilepsy [79].

### 5.2. Prognostic Value in Newly Diagnosed Seizure Disorders

When evaluating the first EEG, the clinical context and diagnosis must be taken into consideration. For example, patients with Rett syndrome, a genetic disorder related to *MECP2* mutations, have central IEDs on EEG as part of their condition, but may not have seizures (Figure 2c) [84]. Conversely, young children with runs of 3 Hz generalized spike-wave discharges are likely experiencing absence seizures. However, the presence and characteristics of IEDs at diagnosis can provide valuable insights into the likelihood of achieving seizure control and can inform initial treatment strategies. Approximately one-third of patients with new-onset seizures exhibit IEDs on their initial EEG, with marginally more abnormalities in patients presenting with new-onset epilepsy than patients presenting with the first unprovoked seizure [85]. For those with normal initial EEGs, a subsequent recording following sleep deprivation can reveal abnormalities in an additional third of patients. Approximately 40% to 50% of patients presenting with a first unprovoked seizure will have a recurrence within the subsequent 2 years [86]. The detection of IEDs significantly increases the risk of seizure recurrence, particularly among individuals with unknown or undetermined causes of epilepsy. Population-level trends show that the presence of IEDs is associated with seizure recurrence rates from approximately 35% to 65% in children and from 27% to 50% in adult epilepsy [87,88].

### 5.3. Prognostic Value and Clinical Implications of EEG Monitoring

The presence of IEDs on EEG monitoring of patients with ongoing epilepsy is also clinically useful. A study observed that IEDs significantly increase following anti-seizure medication withdrawal, particularly before and after seizure occurrence [89]. These findings reinforce the idea that IEDs reflect underlying cortical excitability associated with an elevated risk of seizures. As such, tracking IED frequency at regular circadian intervals could provide insight into an individual’s seizure susceptibility.

Additionally, IEDs observed on routine EEG in patients with epilepsy may influence the risk of future seizures. IEDs on follow-up EEG independently predict increased seizure risk, especially in patients with generalized epilepsies [90]. These findings suggest that IEDs on routine EEG can predict seizure recurrence and inform clinical management during epilepsy follow-up.

### 5.4. Activation Procedures and IEDs

Hyperventilation (HV) is currently used in standard practice to assist with the diagnosis of epilepsy during EEG recording [91]. The mechanism linking HV to neuronal hypersynchronous spiking activity involves changes in arterial partial pressure of CO_2_ (pCO_2_), decreased cerebral blood flow velocity, and respiratory alkalosis. These physiological changes decrease membrane stability in neuronal tissue, making neurons more easily excited with a tendency toward spontaneous discharges [92]. Both animal models and clinical studies demonstrate that elevated pH increases neuronal excitability and may produce epileptiform activity [93,94]. While HV is well known to activate IEDs in genetic generalized epilepsies [95], it can also activate focal IEDs, though less frequently [96]. In a combined pediatric and adult study, patients with temporal lobe epilepsy appear to be more susceptible to HV activation than those with other focal epilepsies [97].

A study of patients aged 16 to 50 years showed that HV triggered seizures in 23% of those with medically intractable focal epilepsies during video EEG monitoring. The HV-activated seizures resembled spontaneous events and helped shorten the time for presurgical evaluation [97]. HV has even been used intraoperatively to precipitate seizures in an 11-year-old patient with temporal lobe epilepsy [98]. A small study using single photon emission computed tomography in 12 patients aged 23 to 64 years found that lateralized IEDs in focal epilepsy correlated with increased cerebral perfusion in seven of eight patients who experienced activation, suggesting that spiking activities may reflect the pathophysiology of the epileptogenic area [99]. In patients with focal epilepsy, the location of IEDs serves as an important indicator of the cortical area from which seizures originate or propagate.

Emerging evidence suggests a potential link between HV-induced focal seizures and autoimmune encephalitis in adults. A review of approximately 10,000 consecutive routine EEG studies identified seven recordings in six patients with focal HV-induced seizures, each of temporal lobe onset. All patients were diagnosed with autoimmune encephalitis, in two cases following EEG finding, and five had voltage-gated potassium channel complex autoantibodies [100]. Similarly, another study observed HV-induced seizures in 8 of 30 adult patients with autoimmune epilepsy during video-EEG monitoring [101]. Less information is currently available regarding HV activation in children with autoimmune epilepsy.

Photoparoxysmal responses (PPRs) are defined as IEDs associated with intermittent photic stimulation (IPS) or other visual stimuli of everyday life [102,103]. The prevalence of photosensitivity depends on the methods of photic stimulation and definition of PPR; in epileptic patients, however, it ranges from 2 to 9.9% [104,105], and the prevalence of photosensitive epilepsy is 1 in 4000 [106]. The prevalence of photosensitivity in nonepileptic individuals ranges from 0.5 to 8.9% of the population [107,108] and is higher around the age of puberty and more prevalent in females [109,110]. It is important to emphasize that PPRs in patients without seizures rarely evolve into epilepsy [107]. PPRs that persist after cessation of the stimulus may have predictive implications for epilepsy development [111,112].

PPRs can present in various forms, including spikes within the occipital rhythm, parieto-occipital spikes with biphasic slow waves, parieto-occipital spikes with biphasic slow waves spreading to the frontal region, and generalized spike-wave or polyspike-wave discharges [113].

Photic stimulation can provoke IEDs and seizures predominantly in generalized epilepsies, such as juvenile myoclonic epilepsy, but can also occur in focal epilepsies, especially occipital lobe epilepsy, including photosensitive occipital lobe epilepsy (POLE) [114,115,116]. Rarely, PPRs are observed in patients with temporal lobe epilepsy, often accompanied by other focal seizure features [117,118]. The prognosis for controlling photic stimulation-induced seizures is generally favorable [119,120,121].

Notably, PRR to low-frequency intermittent photic stimulation are characteristic EEG findings in certain progressive myoclonic epilepsies, including neuronal ceroid lipofuscinoses, particularly CLN2, CLN5, and CLN6 subtypes. This phenomenon is especially prominent during the early stages of disease [122].

### 5.5. Prognostic Value During Anti-Seizure Medication Discontinuance 

EEG assessment during anti-seizure medication discontinuation involves several important considerations. Key factors include the underlying etiology, epilepsy syndrome, current medication effects, neurological examination findings, and the inherent sensitivity limitations of routine scalp EEG [123].

However, certain interictal EEG abnormalities help identify epilepsy with a poor prognosis for remission and may inform anti-seizure medication tapering decisions. For example, anterior temporal IEDs suggest the diagnosis of temporal lobe epilepsy, which has a high rate of medical intractability and a relatively low remission rate [124]. Other IEDS associated with less favorable outcomes include generalized slow spike-wave, frontal IEDs in frontal lobe epilepsy, and generalized polyspike and wave discharges [123,125,126].

### 5.6. Pre-Surgical Evaluation (IED Associated with High Frequency Oscillations)

High-frequency oscillations (HFOs) are transient oscillations occurring within the broad 80–600 Hz frequency range. This range is typically divided into ripples (80–250 Hz) and fast ripples (250–600 Hz) [127]. HFOs are most often characterized by at least four oscillations that clearly stand out from the background [127]. Since their discovery, HFOs have been shown to be associated with epileptogenicity. For example, studies in models of status epilepticus (with frequency rates of 100–500 Hz) and traumatic brain injury (with frequencies between 100 and 600 Hz) have demonstrated that all animals exhibiting HFOs within two weeks post-insult developed recurrent seizures, while those without HFOs remained seizure-free [128,129]. Initially detected via intracranial electrodes, ripples have subsequently been observed in scalp EEG and MEG recordings [130,131], with fast ripples recorded in scalp EEG of young children with epilepsy [132].

Notably, IEDs co-occurring with HFOs represent a more pathological subtype with greater specificity for the epileptogenic zone, offering important advantages for surgical planning in drug-resistant epilepsy [133,134]. Also, as physiological ripples are rare on scalp EEG, and most frequently at the time of IEDs, they are considered more pathological [135]. It has been shown that at the cellular level, HFO-bearing IEDs demonstrate higher neuronal firing rates, pronounced pre-peak firing increases, and selective neuronal participation, indicating they are highly pathological IEDs [136].

Although fast ripples represent the most accurate biomarker, they are detected less frequently in patients with drug-resistant epilepsy [137,138,139]. In one study of 40 children undergoing intracranial monitoring, spikes-on-ripples were observed in all patients, whereas fast ripples were detected in only half [140]. Patients with favorable outcomes demonstrated higher rates of both fast ripples and spikes-on-ripples within the resected area. While fast ripples achieved the highest accuracy (82%) in predicting surgical outcome, spikes-on-ripples demonstrated superior precision (positive predictive value of 90%) [140]. A large multicenter cohort found that successful surgical resection removed the majority of spike-ripples, and that automatically detected spike-ripples localized epileptogenic tissue more effectively than spikes alone, spike-gamma, wideband HFOs, ripples, or fast ripples [141]. Additionally, spike-ripple onset overlap (SRO) zones, where spike and ripple onsets coincide, serve as specific and precise biomarkers of the epileptogenic zone, with their complete removal predicting favorable surgical outcome [142]. Studies on scalp EEG have also shown that ripples-on-spikes are prognostic, non-invasive biomarkers of epileptogenicity and that removing their cortical generators predicted good surgical outcome [143].

### 5.7. Prognostic Value After Epilepsy Surgery

Patients with refractory focal epilepsy may undergo surgery to control seizures. The Engel Epilepsy Surgery Outcome Scale is widely used to classify outcomes after surgical treatment for medically refractory epilepsy. Successful epilepsy surgery corresponds to Engel Class I, where patients are free of disabling seizures after surgery. The most successful outcomes include Engel IA, where patients are completely seizure-free, or Engel IB, where patients experience only non-disabling focal aware seizures since surgery [144]. However, approximately one-third of those with successful surgery experience seizure recurrence after discontinuing anti-seizure medications [145]. The persistence of IEDs postoperatively may indicate incomplete resection of the epileptogenic zone and a higher likelihood of seizure recurrence.

Prospective studies demonstrate a strong association between postoperative IEDs and increased risk of seizure recurrence in successfully operated patients undergoing anti-seizure medication withdrawal [146,147]. Specifically, the presence of IEDs one year after surgery elevates the risk of seizure recurrence in patients with both mesial temporal lobe epilepsy and lesional extratemporal epilepsy. Contralateral temporal IEDs are associated with less favorable surgical outcomes after temporal lobectomy [148]. Notably, in individuals with disconnection surgeries, such as a hemispherectomy, post-operative EEG abnormalities do not consistently predict outcome [149].

## 6. Conclusions

Although IEDs are widely acknowledged for their clinical significance, IEDs do not possess equal prognostic value. Variations in IED topography, morphology, frequency and timing have important implications for prognosis in pediatric epilepsy. As the brain matures from neonates to older children, the location and characteristics of IEDs evolve, thereby influencing their prognostic relevance. Certain IED locations, such as midline, are associated with higher epileptogenic potential and neurodevelopmental issues. IED morphological differences also impact clinical outcome. In addition to SeLECTs, IEDs exhibiting tangential dipoles, regardless of their brain regions, are typically linked to a more benign course. Conversely, the presence of positive sharp waves, beyond the neonatal period, may indicate a less favorable prognosis. IEDs may have an impact on cognition in children. Specifically, IEDs that are markedly potentiated during slow-wave sleep have been associated with cognitive regression in some cases. The timing of IED occurrence, such as at the time of seizure onset, treatment, anti-seizure medication withdrawal, or surgical intervention, also holds significant prognostic value. Recognizing these nuanced differences in focal IEDs enhances our ability to predict clinical outcomes, tailor treatment strategies, and optimize long-term management for children with epilepsy.

## 7. Future Directions

Most of the studies in this review utilized scalp EEGs and IED characteristics based on visual analysis. However, the use of the 10-20 EEG system limits scalp coverage and delineation of deeper brain activity. Future studies with prolonged video-EEG monitoring, sequential topographical mapping, high-resolution recordings from electrode arrays, magnetoencephalography and detection of high-frequency oscillations and spikes with ripples on pediatric intracranial and scalp EEG could yield further insights. Although some studies included cohorts that underwent standardized EEG recording protocols in the laboratory, this was not consistent across all studies. The development of standardized EEG databases across institutions would significantly enhance our understanding of IEDs. The studies included in this review exhibited considerable methodological heterogeneity, with variations in age groups (ranging from neonates to young adults), EEG recording durations, and sleep recording protocols.

This review provides an overview of the existing literature; however, it is not a comprehensive systematic review or meta-analysis. Moreover, most of the studies included in this review primarily focused on scalp EEGs conducted at tertiary care centers, which may limit the generalizability of the findings to the broader population of patients undergoing EEG evaluation. Prospective multicenter studies with formal neuropsychological evaluations will further enhance the prognostic significance of IEDs on routine EEG. Further clarification of cortical connectivity will enhance our understanding of the relationship between neuronal structure and clinical symptomatology.

## Data Availability

The original contributions presented in this study are included in the article. Further inquiries can be directed to the corresponding author(s).
